# Glaucocalyxin A delays the progression of OA by inhibiting NF-κB and MAPK signaling pathways

**DOI:** 10.1186/s13018-024-04640-z

**Published:** 2024-03-18

**Authors:** Xin Hong, Xuqiang Liu, Bo Li, Shoujie Shi, Kai Xiao, Tiantian Xu, Yaoyang Nie, Min Dai, Meisong Zhu

**Affiliations:** 1 Orthopedic Hospital, The First Affiliated Hospital, Jiangxi Medical College, Nanchang University, Jiangxi Province’s Artificial Joints Engineering and Technology Research Center, Nanchang, 330006 Jiangxi Province China; 2 Department of Pharmacy, The First Affiliated Hospital, Jiangxi Medical College, Nanchang University, Nanchang, 330006 Jiangxi Province China

**Keywords:** OA, Glaucocalyxin A, NF-κB, MAPK, Extracellular matrix

## Abstract

**Background:**

Osteoarthritis (OA) is a common degenerative joint condition marked by inflammation and cartilage breakdown. Currently, there is a dearth of treatment medications that can clearly slow the course of OA. Glaucocalyxin A (GLA) is a diterpene chemical identified and extracted from Rabdosia japonica with antithrombotic, anticoagulant, anti-tumor, anti-inflammatory, anti-oxidant, and other pharmacological properties. Previous research has linked inflammation to abnormalities in the homeostasis of the extracellular matrix (ECM). Although GLA has been shown to have anti-inflammatory qualities, its effects on the progression of OA are unknown. As a result, the goal of this study was to see if GLA could slow the course of OA.

**Methods:**

ATDC5 cells were stimulated by IL-1β to create an inflammatory chondrocyte damage model. Quantitative polymerase chain reaction, Western Blot, high-density culture, and immunofluorescence were used to detect the expression levels of associated gene phenotypes. We also created a mouse model of OA induced by destabilization of the medial meniscus (DMM) instability, and GLA was administered intraperitoneally once every two days for eight weeks. Mice knee specimens were stained with hematoxylin–eosin, Safranin O/fast green, and immunohistochemical, and the Osteoarthritis Research Society International grade system and Mankin’s score were used to assess the protective effect of GLA on cartilage.

**Results:**

In vitro and in vivo, we explored the effects and molecular processes of GLA as a therapy for OA. The findings demonstrated that GLA might reduce the expression of associated inflammatory mediators and protect the ECM by inhibiting the NF-κB and MAPK signaling pathways. Animal research revealed that GLA could protect against the DMM-induced OA model mice by stabilizing ECM.

**Conclusion:**

Taken together, our findings show that GLA has a protective impact on cartilage throughout OA progression, implying that GLA could be employed as a possible therapeutic agent for OA, thus giving a new therapeutic method for the treatment of OA.

## Introduction

Osteoarthritis (OA) is a common joint condition that can cause persistent pain, joint stiffness, and impairment in the aged. It is a disease that degenerates mainly involving arthroidal cartilage, ligament, subchondral bone, synovium, joint capsule and muscle structure around the joint [1]. Although there is still controversy about whether the process of OA begins with articular cartilage, calcified cartilage or subchondral bone, the loss of articular cartilage is a prominent feature in the evolution of OA, and almost all joints have the loss of articular cartilage in the evolution of OA, which is now widely believed [2]. Chondrocytes, as the only cell type of articular cartilage, maintain the stability of articular cartilage matrix components in a normal in vivo environment. In the abnormal in vivo environment, chondrocytes are exposed to the abnormal environment, and inflammatory cytokine genes are activated and secreted in large quantities. Chondrocytes will change from stable to catabolic phenotype, leading to degeneration of articular cartilage [3].

Among these cytokines, interleukin-1β (IL-1β) has been shown to play an essential role in the development of OA [4]. IL-1β activates the nuclear factor-kappa B (NF-κB)/mitogen-activated protein kinase (MAPK) signaling pathway, resulting in excessive production of nitric oxide (NO) and prostaglandin E2 (PGE2), resulting in enhanced gene expression of inflammatory markers cyclooxygenase-2 (COX-2), inducible nitric oxide synthase (INOS), and hypertrophic marker matrix metalloproteinase 13 (MMP13), resulting in articular cartilage destruction and joint pain [5–7]. In addition, IL-1β also inhibited the expression of cartilage formation markers SRY box gene-9 (SOX9) and type II collagen (COL2A1). Previous studies have shown that the ECM is primarily made up of COL2A1, which is essential for maintaining the dynamic equilibrium of joint tissue, and SOX9 overexpression can stimulate cartilage repair. Therefore, the inhibition of COL2A1 and SOX9 expression will inevitably aggravate the formation of OA [8]. Therefore, the release of inflammatory factors is thought to be closely linked to OA formation and development. Inhibiting the inflammatory response generated by IL-1β could be an effective therapy for OA.

Natural chemicals have grown in popularity in recent years due to their low risk of negative effects. *Rabdosia japonica* (Burm. f.) Hara var. *glaucocalyx* (Maxim.) Hara is a perennial herb that is commonly grown throughout East Asia. This plant's above-ground growing parts are widely utilized in traditional medicine for its anti-inflammatory, antibacterial, anti-cancer, and anthelmintic properties [9]. Glaucocalyxin A (GLA pubchem CID: 10471963) is an active component prevalent in *R. japonica's* aboveground section [10, 11].

According to the existing studies, GLA has multifarious pharmacological activities such as antioxidant, antithrombotic, anticoagulant, anti-tumor, anti-inflammatory and so on [12, 13]. According to previous studies, GLA can inhibit epithelial-mesenchymal transition in osteosarcoma by restraining TGF-β1/Smad2/3 signal transduction pathway, so as to achieve the effect of treating osteosarcoma [14]. In addition, GLA can also protect rat cardiomyocytes from hypoxia injury by activating Akt/Nrf2/HO-1 pathway [15]. In terms of anti-inflammation, studies have shown that GLA can reduce airway hyperresponsiveness and secretion of inflammatory cells in asthmatic mice, inhibit the proliferation of goblet cells, and decreasing the expression level of IL-4, IL 5, and IL-13 [16]. GLA can also attenuate endotoxin-induced septic shock and aggravation by decreasing NLRP3 inflammasome activation [17]. Given GLA’s anti-inflammatory action, and the fact that the mediation of inflammatory effects plays a critical part in the onset and progression of OA, we have reason to believe that GLA could be a possible anti-inflammatory alternative for the treatment of OA. The goal of this study was to look into the influence and biochemical mechanisms underlying GLA during the therapy of OA in vitro and in vivo.

## Materials and methods

### Chemicals and reactants

**Fig. 1:**
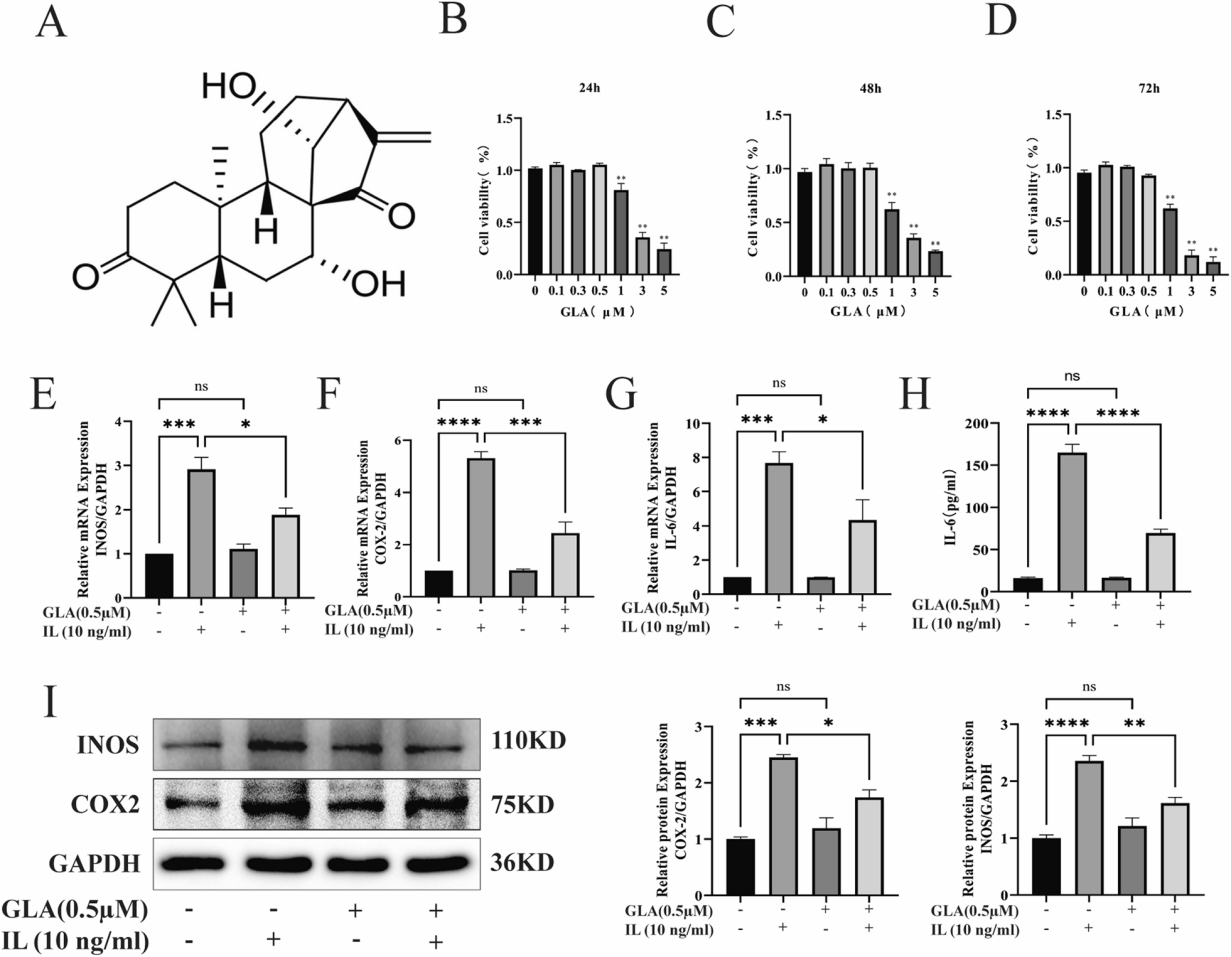
Glaucocalyxin A’s chemical structure and cytotoxic action on mouse chondrocytes. GLA can inhibit the production of inflammation-related factors when stimulated by IL-1β. ATDC5 cells activated or unstimulated by 10 ng/ml IL-1β were cultured for 2 days with or without GLA (0.5 µm). Western blot and qPCR were used to further examine the isolated RNA and total protein. **A** GLA’s chemical structure. **B**–**D** was utilized to access the cytotoxicity of different GLA concentrations and times on chondrocytes. **E**–**G** GLA decreased the expression of inflammatory cytokine-related marker genes. **H** The production of IL-6 in chondrocyte culture supernatant was detected using a double antibody sandwich technique. **I** Western blot analysis was employed to determine iNOS and COX-2 protein expression. The reported value is the mean SD of three independent experiments. ***p < 0.001, **p < 0.01, *p < 0.05, when compared to the control group

Shanghai Yuanye Bio-Technology Co., Ltd (Shanghai,China) provided GLA (CAS No. 79498-31-0, Batch No. B20698, 98% purity). The chemical structure of GLA is illustrated in Fig. 1A. To obtain the original solution (50 mM), we dissolved the GLA in dimethyl sulfoxide and kept it at − 80 °C. Pancreatic enzyme, Dulbecco’s Modified Eagle Media: Nutrient Mixture F-12 (DMEM/F12), radio-immunoprecipitation assay (RIPA) buffer, 4, 6-diamidino-2-phenylindole (DAPI) solution and toluidine blue staining solution were purchased from Beijing Solaybao Technology (Beijing, China). Betulinic acid (Batch No. S360301, CAS No. 472-15-1, 99.88% purity) was purchased from selleck (Shanghai, China). Horseradish peroxidase-conjugated goat anti-rabbit and goat anti-mouse antibodies were purchased from Boster Biological Technology (Wuhan, China). Primary antibodies against Primary antibodies against (GAPDH), COL2A1, MMP13, SOX9, iNOS, COX-2 were obtained from Abcam (Shanghai, China). Primary antibodies against extracellular signal-regulated kinase (ERK), c-Jun N-terminal kinase (JNK), NF-κB p65, phosphorylated-ERK (p-ERK;Thr202/Tyr204),phosphorylated-JNK (p-JNK;Thr183/Tyr185), phosphorylated-NF-κB p65 (p-P65;Ser536) were from Cell Signaling Technology (Danvers, MA, USA).

### Cell culture and grouping

A chondrogenic ATDC5 cell line was purchased from Riken Cell Bank (Ibaraki, Japan) and was used as a reference for the analysis. ATDC5 cells were grown at 37 °C in a cell incubator with 5% carbon dioxide in DMEM/F12 media supplemented with 10% fetal bovine serum and 1% penicillin/streptomycin. For control experiments, ATDC5 cells were divided into four groups. To observe the GLA in IL-1β stimulation cartilage cells play a role. (1) Sham group, GLA and IL-1β were not administered to the ATDC5 cells; (2) IL-1β group, In the case of ATDC5 cells, only IL-1β (10 ng/ml) was used; (3) GLA group, Only the GLA (0.5 μM) was applied to the ATDC5 cells; (4) GLA+IL-1β group, we treated ATDC5 cells with IL-1β (10 ng/ml) and GLA (0.5 μM). GLA is dissolved in DMSO. In this experiment, the concentration of DMSO in all cell culture media was kept below 0.05%, and there was no significant effect on the growth of cells.

### Cell-viability assay

CCK-8 reagen was used to detect the effect of GLA on chondrocyte cell viability. ATDC5 cells (6 × 10^3^ cells/well) were planted in 96-well plates and cultivated for 24 h. ATDC5 cells then underwent treatment with GLA at various doses (0, 0.1, 0.3, 0.5, 1, 3, 5 μM) during 24 h, 48 h, or 72 h. Following treatment, 10μl CCK-8 solution was added into each well and it was incubated at 37 °C for 1–4 h. Samples were assayed for absorbance at the 450 nm band level using an ELX800 microplate reader (Bio-Tek Instruments, Inc, Winooski, VT, USA). All experiments were carried out independently three times.

#### RNA extraction and quantitative reverse transcription PCR

ATDC5 cells were separated into four groups and injected in 6-well plates (1 × 10^5^ cells/well). The cells were treated for 48 h with IL-1β or GLA, and total RNA from chondrocytes in each of the groups was isolated with TRIzol (Beijing Tianen Biotechnology Co., Ltd., Beijing, China). We took RNA (1 μg) from each chondrocyte group and synthesized complementary Dna using reverse transcriptase according to the manufacturer’s protocol (TaKaRa Bio, Otsu, Japan). We performed real-time PCR using SYBR PreMix Ex Taq Kit (Takara Bio) and a LightCycler 96 PCR machine (Roche, Germany). All reactions were carried out in triplicate. The specific primers employed are as follows:

INOS Forward primer 5′-CTGGCAAGCCCAAGGTCTAT-3′

Reverse primer 5′-TCCCCGCAAACATAGAGGTG-3′;

COX-2 Forward primer 5′-CGG TGAAACTCTGGCTAGACAG-3′

Reverse Sequence 5′-GCAAACCGT AGATGCTCAGGGA-3′;

MMP13 forward primer 5ʹ-GACCCCAACCCTAAGCATCC-3′

Reverse primer 5ʹ-CCTCGGAGACTGGTAATGGC-3′;

COL2A1 forward primer 5′-CTCAAGTCGCTGAACAACCA-3′

reverse primer 5′-GTCTCCGCTCTTCCACTCTG-3′;

SOX9 forward primer 5′-GCAGGCGGAGGCAGAGGAG-3′

reverse primer 5′-GGAGGAGGAGTGTGGCGAGTC-3′;

GAPDH forward primer 5′-ACCCAGAAGACTGTGGATGG-3′

reverse primer 5′-CACATTGGGGGTAGGAACAC-3′.

#### Western blot analysis

We washed the ATDC5 cells twice with cold PBS after treating them with IL-1β or GLA, and then extracted the total cellular proteins from the ATDC5 cells using RIPA lysis solution mixed with protease and phosphatase inhibitors (Sigma-Aldrich, Rockford, USA). The lysate was kept on ice for 10 min before centrifuge at 12,000 rpm for 10 min at 4 °C. The required total protein was present in the supernatant. The target protein quantity was evaluated using a Bicinchoninic Acid (BCA) Protein assay kit (Beyotime, Nanjing, China), and the protein was quantified. Sodium dodecyl sulfate–polyacrylamide gel (SDS-PAGE) was used to electrophorese protein (20 μg/well), which was then transferred to polyvinylidene fluoride membranes (0.45 μm, Millipore, Bedford, MA, United States). The polyvinylidene fluoride (PVDF) membrane has been blocked with 5% skimmed milk for 2 h at ambient temperature before being treated with the matching primary antibody overnight at 4 °C. The PVDF membrane was washed three times with Tris-buffered saline-Tween 20 for ten minutes each time before being incubated with a horse-radish peroxidase (HRP) conjugate secondary antibody for one hour at ambient temperature. Finally, we detected the protein bands using the Odyssey V3.0 image scanner (LiCOR Biosciences, Lincoln, NE, USA) and analyzed them using ImageJ software. In this experiment, GAPDH was used as a source of internal control.

### Enzyme-linked immunosorbent assay (ELISA)

ATDC5 cells have been grown on 6-well plates, and their supernatant was collected following treatments. The enzyme-linked immunosorbent assay kit (ELISA; R&D System) was used to measure the quantity of IL-6 in the supernatant.

### Immunofluorescence microscopy

ATDC5 cells were seeded in 24-well plates and exposed to either IL1β or GLA for 48 h. For 30 min, the cells were submerged in 4% paraformaldehyde (PFA) at the ambient temperature. For 15 min, the cells were treated with 0.1% Triton-100 (Solarbio, Beijing, China). The primary antibody was incubated at 4 °C overnight after blocking with 1% bovine serum albumin (Sigma Aldrich, Germany) for 30 min. The cells were rinsed three times with PBS before being incubated at room temperature for two hours with goat anti-rabbit Ig G antibody (1:400). After that, the nucleus had been stained for 5 min using DAPI staining buffer. Under a confocal microscope (Leica, Germany), cells are viewed and photographed.

### High density culture and toluidine blue staining

We used this method to measure chondrocyte ECM level. Each well of a 24-well plate was seeded with 10 μL of ATDC5 cells suspension (10^7^ cells), and each group was seeded three times. ATDC5 cells were grown for 1 h in a cell incubator until they stuck to the wall. After cell adhesion, for a total of 24 h, each well received 700 μL of DMEM/F12 medium for cell cultivation with a concentration of 10% fetal bovine serum and 1% penicillin/streptomycin. Then different quantities of IL-1β (10 ng/ml) and GLA (0, 0.3, 0.5 μM) were added. Every 2–3 days, the culture media was replaced. After 5–8 days of cell culture, ATDC5 cells were fixed for 30 min in 4% PFA before being stained with the color toluidine blue. ImageJ (National Institutes of Health, Bethesda, MD, United States) was used to determine the staining intensity of cells in each group.

### Animal model

All experiments involving animals in the present study have been carried out in conformity with Nanchang University’s Animal Ethics Committee (No.: DM20210912). The animal model we established this time is the DMM model [18]. C57BL/6 mice (6 weeks old, n = 24) were anesthetized with a 50 mg/kg intraperitoneal dose of pentobarbital sodium. Under the microscope, the attachment point between the medial meniscus and the tibial plateau of the right knee (medial meniscus-tibial ligament) was severed with a surgical knife, and the injury of other ligaments around the medial meniscus should be avoided during the operation. The experimental animals were randomly divided into 4 groups: Sham group, DMM group, DMM + 10 mg/kg GLA group (low concentration GLA group), DMM + 20 mg/kg GLA group (high concentration GLA group). Only the right knee joint was incised in the Sham group, without the medial meniscus-tibial ligament being severed. The mice in the low-concentration GLA group were intraperitoneally injected with GLA 10 mg/kg, and the mice in the high-concentration GLA group were given GLA 20 mg/kg intraperitoneally on two separate days for 8 weeks. Mice in the sham surgery and DMM groups received the same quantity of phosphate buffered saline (PBS) intraperitoneally. During the experiment, mice were free to obtain food and water. After 8 weeks, pentobarbital sodium was injected intraperitoneally. Following anesthesia, each of the mice were euthanized, and samples of knee tissue from the joints were taken for further research.

#### Histological scoring

Six mice in each group had their right knee joints frozen in 4% paraformaldehyde for 24 h before being dissolved with a 10% ethylenediaminetetraacetic acid (EDTA) decalcified solution for one month. The mice’s knee joints were implanted in paraffin, and the knee joints containing the mice were cut into sections of 5 μm thickness from the sagittal position. Hematoxylin–eosin (HE) staining, safranin O-fast green staining and IHC staining (p-p65, MMP13, COL2A1, COX-2) were performed on the sections. In this study, the pathological changes of joints were analyzed by the International Association for the Study of OA (OARSI) scoring system [19]. Xylene was used to dewax the paraffin sections of the knee joint, and the related staining was performed after gradient ethanol hydration. Under a microscope, the cartilage damage of the joint in the knee and the manifestation of associated indicators were seen.

### Statistic analysis

All results were shown as mean ± standard deviation (N ≥ 3). For statistical analysis, we utilized GraphPad Prism 9 software (GraphPad Software, California, USA). To examine the variations between two groups or three or more groups, all statistical analyses were performed using the t-test or a one-way analyses of variance (ANOVA). In statistical terms, *P* 0.05 was considered meaningful.

## Results

### Effects of GLA on chondrocyte cell viability

In this experiment, we used CCK-8 method to detect the potential cytotoxic effect of GLA on chondrocytes. ATDC5 cells received treatment for 24 h, 48 h, and 72 h with various doses of GLA (0, 0.3, 0.5, 1, 3, and 5 μM). GLA exhibited no cytotoxicity to ATDC5 cells at concentrations ranging from 0 to 0.5 μM (Fig. 1B–D). Therefore, 0.3 μM and 0.5 μM concentrations of GLA were used as experimental concentrations in subsequent studies.

### GLA suppressed inflammatory factor release by decreasing IL-1β and lowered the levels of INOS and COX-2

To study the impact of GLA in an IL-1β-induced chondrocyte inflammation paradigm, we first treated ATDC5 cells with GLA (0.5 μM) for 2 h before stimulating them with or without IL-1β (10 ng/ml) for 48 h. The expression of iNOS and COX-2 in cells was detected by qPCR and WB. In addition, we also employed ELISA to assess the level of IL-6 expression in chondrocyte culture supernatant. The qPCR analyses demonstrated that, as compared with the untreated group, in ATDC5 cells, GLA had no influence on the levels of iNOS, IL-6, or COX-2, while IL-1β mediated ATDC5 cells significantly enhanced the expression of iNOS, IL-6 and COX-2 (Fig. 1E–G). The data results of WB also illustrate this point (Fig. 1I). In addition, we also utilized ELISA to see how GLA affected IL-1β-induced IL-6 production in ATDC5 cells. The findings revealed that IL-6 release increased after IL-1β activated ATDC5 cells. After treatment of IL-1β-mediated ATDC5 cells with GLA, the content of IL-6 in the cell supernatant was significantly reduced (Fig. 1H).

### The regulatory effect of GLA on the production of cartilage extracellular matrix and cartilage markers

**Fig. 2:**
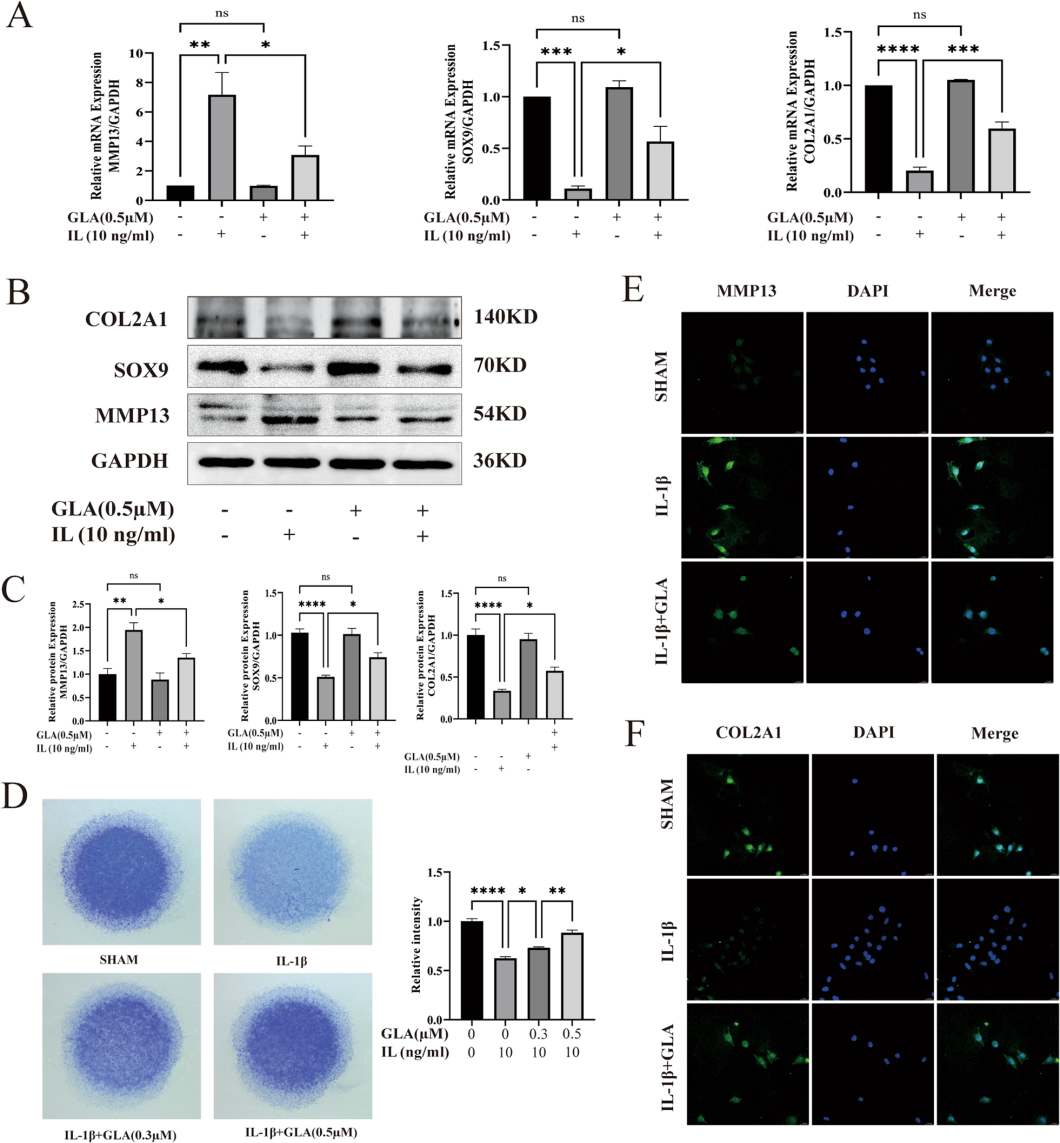
GLA can prevent IL-1β-induced cartilage extracellular matrix breakdown. **A** qPCR study of mRNA expression of MMP13, SOX9, and COL2A1. **B** &** C** MMP13, SOX9, and COL2A1 protein expression levels were determined by Western blot. **D** IL-1β stimulated cells were seeded at a cell density of 10^7^/ml in 24-well plates and treated with or without GLA for 6 days. This is a stained cell picture using toluidine blue solution.** E** &** F** MMP13 and COL2A1 immunofluorescence (ration: 20 µm). The reported value is the mean SD of three independent experiments. ***p < 0.001, **p < 0.01, *p < 0.005, when compared to the control group

The purpose of this study was to see how GLA affected the extracellular matrix and cartilage markers of chondrocytes generated by IL-1β. We used qPCR, WB, toluidine blue staining and IF to detect the expression of extracellular matrix and cartilage markers secreted by chondrocytes. The results of qPCR indicated that IL-1β could significantly up-regulating the level of MMP13 expression and decrease the expression level of COL2a1 and SOX9. GLA significantly downregulated MMP13 and upregulated COL2a1 and SOX9 expression levels in IL1β-induced ATDC5 cells (Fig. 2A). The results of WB and qPCR results are basically identical (Fig. 2B, C). From the toluidine blue staining experiment, The ability of ATDC5 cells to secrete extracellular matrix triggered by IL-1β was considerably reduced, and GLA promoted the extracellular matrix secretion of ATDC5 cells induced in a way that is dose-dependent by IL-1β (Fig. 2D). The results of the IF staining demonstrated that IL-1β stimulation significantly reduced the expression of COL2a1 while increased the level of matrix metalloproteinase 13 (MMP13), a hypertrophic marker, in ATDC5 cells generated by IL-1β, which was suppressed by GLA (Fig. 2E, F). The preceding experiment consists of three different repeated experiments.

### GLA inhibited IL-1β-induced NF-κB and MAPK signal transduction pathways

**Fig. 3:**
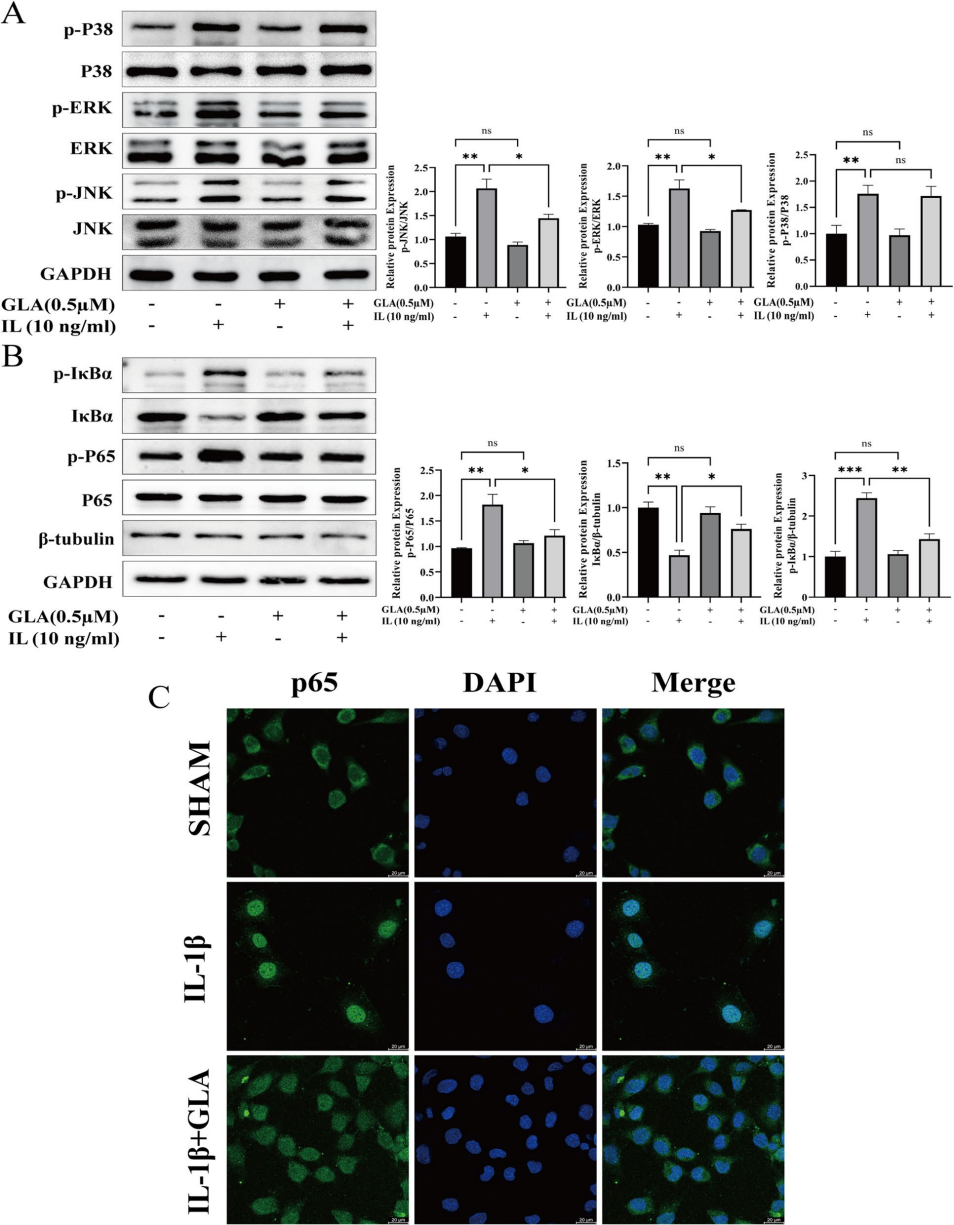
GLA’s effect on NF-κB and MAPK signaling pathways in IL-1β-stimulated chondrocytes. **A** Western blot and quantification analysis were used to detect the expression levels of P38, p-P38, ERK, p-ERK, JNK, and p-JNK. **B** Western blot and quantification analysis were used to identify the expression levels of P65, p-P65, IκBα, and p-IκBα in the NF-κB signaling pathway. **C** Immunofluorescence was used to visualize P65 nuclear translocation. The reported value is the mean ± SD of three independent experiments. ***p < 0.001, **p < 0.01, *p < 0.05, when compared to the control group

NF-κB and MAPK signaling pathways are classic inflammatory pathways [20, 21]. In order to determine whether GLA can directly inhibit NF-κB and MAPK signaling activity, We used WB and IF staining to see if these routes of signaling are involved in GLA's anti-inflammatory effect. IL-1β therapy dramatically increased phosphorylation levels of P38, JNK, and ERK in the MAPK signaling pathway, according to Western blot analysis. GLA did not lower P38 phosphorylation, however it did dramatically reduce JNK and ERK phosphorylation (Fig. 3A). Furthermore, after IL-1β stimulation of ATDC5 cells, IκBα (inhibitor of NF-κB) in ATDC5 cells is phosphorylated to phosphorylated-IκBα (p-IκBα) and subsequently degraded, this causes the NF-κB signaling pathway to be activated, but GLA can inhibit the stimulation of IL-1β on ATDC5. Decreased phosphorylation levels of p65 (Fig. 3B). The results of IF staining also confirmed this: GLA suppressed p65 translocation into the nucleus considerably (Fig. 3C) taking into account the data presented above, we may conclude that GLA lowers inflammatory expression in IL-1β-mediated chondrocytes primarily by blocking the NF-κB and MAPK pathways of signaling.

### Functional recovery experiment

**Fig. 4:**
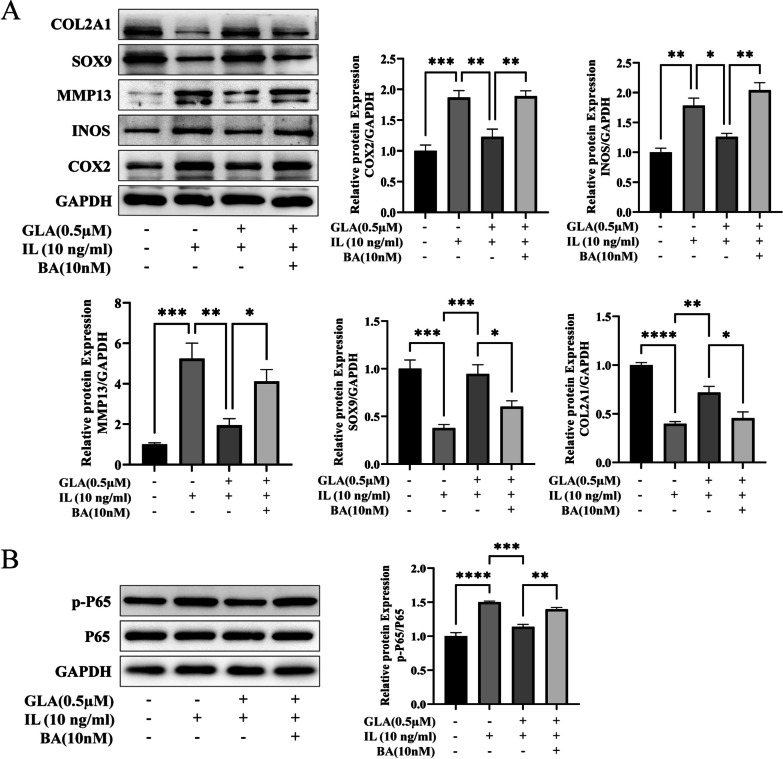
GLA impacted the expression of associated gene phenotypes via the NF-κB signaling pathway, according to functional recovery experiments. **A** Western blot and quantification analysis confirmed that BA, an NF-κB signaling pathway activator, may influence the expression levels of COX2, INOS, MMP13, SOX9, and COL2A1 in chondrocytes. **B** Western blot and quantification analysis cinfirmed that BA, and activator of the NF-κB signaling pathway, may influence p65 and p-p65 expression levels in chondrocytes

To determine if GLA modulates inflammatory expression via blocking the NF-κB signaling pathway, more research is needed. So we used the NF-κB agonist betulinic acid (BA, 10 nM) in vitro [22]. To determine whether GLA modulates the degree of inflammatory expression by decreasing the NF-κB signaling pathway, we isolated total protein from ATDC5 cells and used WB to identify the expression of signaling pathways and associated markers. The Wb results revealed that when BA was added to cells treated with IL-1β and GLA, the level of p-P65 in the cells increased significantly, as did the relative levels of protein expression of INOS, COX-2, and MMP13, while the relative protein expressing themselves levels of SOX9 and COL2A1 decreased (Fig. 4A, B). This finding supported our hypothesis that GLA regulated inflammatory factor production and ECM homeostasis in chondrocytes via suppressing the NF-κB signaling pathway.

### GLA can slow down the progress of arthritis in DMM model of OA mice

**Fig. 5:**
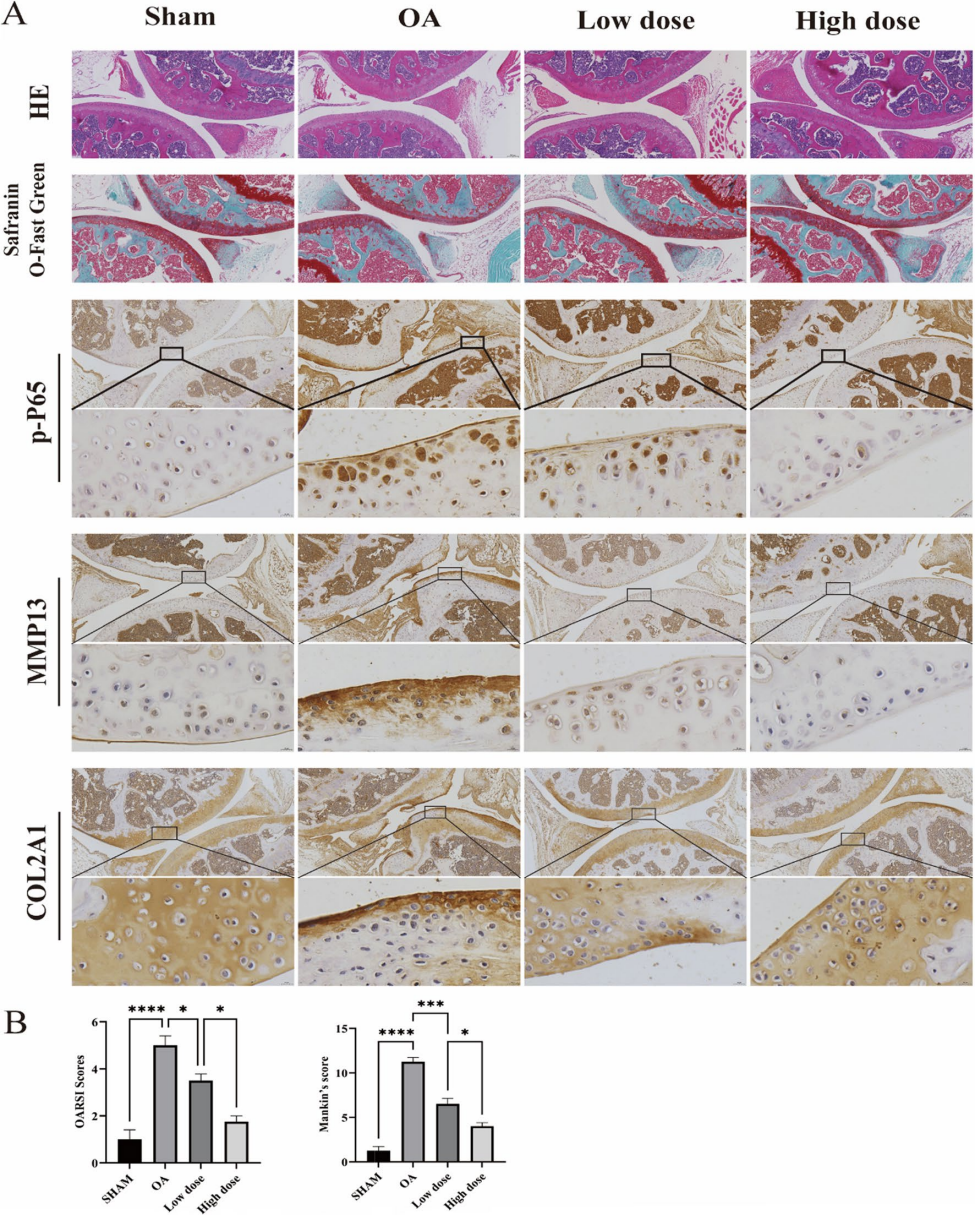
The knee joint of mice was examined histopathologically and immunohistochemically. **A** Representative HE, SO/FG, p-P65, MM13, and COL2A1 staining photos. The photos were shown at two magnifications: low (×10 magnification, scale 100 µm) and high (×96 magnification, scale 10 µm). **B** Glaucocalyxin A has been shown to considerably lower the OARSI and Mankin scores

To investigate the effect of GLA on the genesis and growth of OA in vitro, a DMM-induced OA mice model was created, and GLA was given intraperitoneally to suppress the development of OA. Following modeling, the knee joint specimens had been colored with hematoxylin–eosin (HE) and saffron O/fast green. The staining results revealed that proteoglycan degradation and the DMM group had much more cartilage deterioration than the sham-operated group, and that GLA successfully prevented these processes in OA (Fig. 5A). The results of OARSI score and mankin's score also confirmed that GLA inhibited the development of OA (Fig. 5B). IHC staining revealed that p-P65 and MMP13 expression was dramatically increased, while the expression of COL2A1 was significantly down-regulated in DMM-induced OA mice compared with Sham group. This suggested that GLA could reduce the expression of p-P65 and MMP13 in OA, slow COL2A1 degradation, and maintain the extracellular matrix of cartilage (Fig. 5A). The results of this experiment are basically consistent with those of in vitro experiments.

## Discussion

One of the most frequent joint illnesses is OA. The main symptoms include joint pain, transient morning stiffness and limited joint movement, which can also lead to physical disability in severe cases. Previous research has demonstrated that OA not only had a detrimental effect on the standard of life of patients, but also places financial strain on them [23, 24]. Although the pathogenesis of osteoarthritis is very complex, and many specific pathological mechanisms are not fully understood, there is a lot of evidence indicating chondrocytes and synovium cells release a lot of inflammatory molecules such IL-1β, nitric oxide (NO), and TNF-α during the development of OA [25, 26]. IL-1β can also mediate the production and secretion of other inflammatory mediators, such as iNOS, PGE2, and COX2 [27, 28]. Among them, a variety of inflammatory mediators interact with each other to accelerate the progression of OA. For example, the high expression of iNOS can induce cells to produce excessive NO, MMPs and TNF-α expression can be increased by NO. The overexpression of MMPs can cause ECM breakdown [29, 30]. COX2 can mediate the high expression of PGE2, leading to articular cartilage damage, subchondral bone structural changes and joint pain [31, 32]. This finding implies that cytokines that cause inflammation play a significant part in the development of OA.

GLA, a diterpene compound extracted from natural plants, has shown good anti-inflammatory and antioxidant impact in disease therapy such as atherosclerosis, asthma, and acute kidney injury [16, 33, 34]. However, there has been no research to determine whether GLA has a substantial therapeutic benefit when used for the treatment of OA. Furthermore, the biological function of GLA in the formation and progression of OA is still unknown, so the biochemical mechanism of GLA in IL-1β-induced chondrocytes model and DMM mice OA model must be investigated. In this work, we discovered that GLA might inhibit the mRNA and protein expression levels of COX-2, INOS and MMP13, inhibit the degradation of SOX9 and COL2A1, and maintain the extracellular matrix homeostasis in inflammatory chondrocytes. Toluidine blue staining of ATDC5 cells in a high-density culture indicated that GLA may prevent IL-1β activation of cartilage cells and stabilize their ECM. GLA has a lot of potential for treating OA relying on these experimental outcomes. Subsequently, we studied molecular mechanism by which GLA inhibits drug action during OA development.

**Fig. 6:**
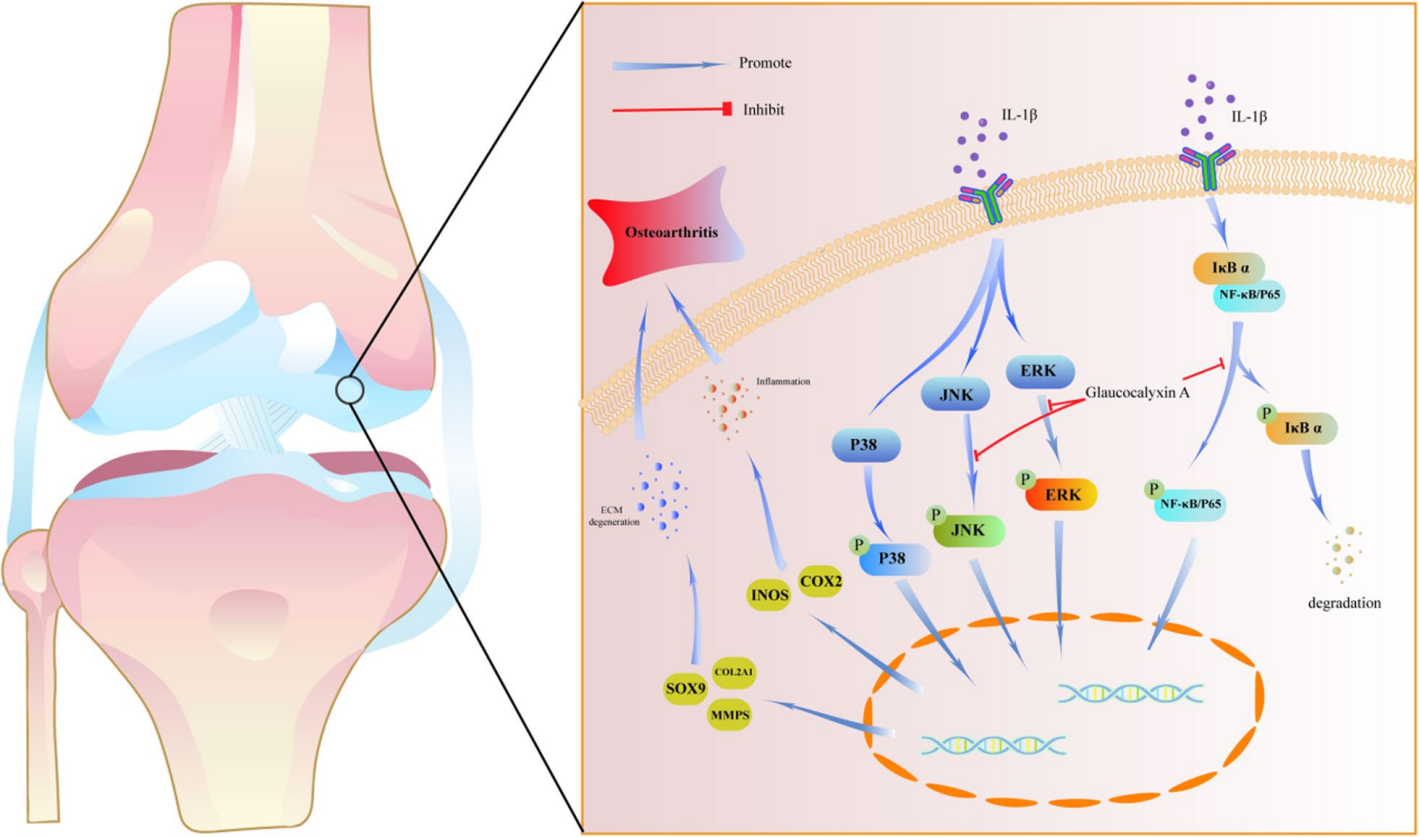
This model demonstrates that GLA is a promising medication for the treatment of OA by blocking the NF-κB, ERK, and JNK pathways in chondrocytes to reduce inflammatory factor production and maintain extracellular homeostasis

Both the NF-κB and MAPK signaling pathways are associated with traditional inflammatory mediators. Under general conditions, NF-κB binds to the inhibitory protein IκBα and the NF-κB signaling pathway is not activated. Nevertheless, in the inflammatory environment stimulated by IL-1β, IκBα is phosphorylated by the activated kinase complex to p-IκBα, which is subsequently dissociated from NF-κB and degraded. In this case, the downstream P65 of the NF-κB signal will be phosphorylated to p-P65 and translocate to the nucleus, As a result, the pathway that communicates with NF-κB is activated, and anti-inflammatory factor expression is regulated [35]. p-JNK/JNK, p-p38/p38, and p-ERK/ERK are all members of the MAPK family. When cells are in an inflammatory environment, MAPK is activated by receiving activation signals from MKKs and MKKKs, P38, ERK and JNK are phosphorylated to p-P38, p-ERK and p-JNK, further activating the MAPK signaling pathway [36, 37]. When these signaling pathways are activated, a large number of inflammatory mediators are produced, such as COX2, INOS and MMP13, which are closely related to OA. So, may we hypothesize that GLA may prevent and treat OA by suppressing the NF-κB and MAPK signaling pathways, which govern the production of associated inflammatory mediators and downstream phenotypes? GLA inhibited the stimulation of the NF-κB signaling pathway by reducing the degradation of IκBα, consequently down-regulating the production of COX-2, INOS, and MMP13. It also reduces the degradation of SOX9 and COL2A1 in inflammatory environment, maintains the stability of ECM and slows the development of OA. In addition,GLA did not suppress P38 phosphorylation. However, by lowering the phosphorylation of JNK and ERK signaling molecules in the MAPK signaling pathway, GLA can prevent the activation of the system and down-regulate the production of related inflammatory markers (Fig. 6).

Given GLA's in vitro anti-OA efficacy, we investigated its influence on a DMM-induced OA mouse model in order to evaluate if GLA has a therapeutic effect on OA animals. The OA mice DMM model dramatically increased p-P65 expression, COX2 and MMP13 expression, and COL2A1 degradation in the cartilage extracellular matrix, according to our findings. GLA can significantly slow the course of OA, minimize cartilage extracellular matrix breakdown, and maintain extracellular matrix homeostasis. Considering the potential organ toxicity caused by systemic administration of GLA during the construction of DMM model in OA mice. We stained the animals' hearts, livers, spleens, lungs, and kidneys with HE. The results showed that no organ toxicity was found, providing preliminary evidence for the safety of GLA in mouse animal experiments. Our findings indicate that GLA, through suppressing the NF-κB and MAPK signal transduction pathways, can limit chondrocyte death and stabilize the cartilage extracellular matrix. The role of GLA was further confirmed by an in vitro IL-1β-stimulated ATDC5 cell model and a DMM-induced mouse OA model.

Of course, this study has some limitations. For example, OA is a complex chronic disease involving the interaction of multiple signaling pathways. Only the NF-κB and MAPK pathways of signaling were researched and investigated in this work; no other putative regulation mechanisms were investigated. Furthermore, this study just looked at the pharmaceutical properties of GLA, and additional pharmacological aspects of GLA that we don’t currently know about need to be investigated further.

## Data Availability

The study's initial contributions have been included in the article/Supplemental Materials; additional questions should be addressed to the corresponding author.

## Abbreviations

GLA: Glaucocalyxin A
ECM: Extracellular matrix
OA: Osteoarthritis
IF: Immunofluorescence
DMM: Destabilization of the medial meniscus
NF-κB: Nuclear factor-kappa B
MAPK: Mitogen-activated protein kinase
INOS: Inducible nitric oxide synthase
MMP13: Marker matrix metalloproteinase 13
COX-2: Cyclooxygenase-2
SOX9: SRY box gene-9
